# Supplementary material for: Complement Factor H and Related Proteins as Markers of Cardiovascular Risk in Pediatric Chronic Kidney Disease
Source: Biomedicines. 2022 Jun 13;10(6):1396. doi: 10.3390/biomedicines10061396 (PMC9220348; doi:10.3390/biomedicines10061396)
Supplement: Supplementary file 1 [file biomedicines-10-01396-s001.zip › biomedicines-1742558-supplementary.pdf]

Table S1. Plasma complement factor H and related protein level in children with CKD.

		CHF μg/ml	CFHR2 μg/ml	CFHR3μg/ml
Etiologies	n			
Renal agenesis	18	751.3(521.2-1049.7)	94.1(75.3-172.2)	47.4(27.9-103.2)
Renal dysgenesis	9	574.6(271.4-1009.2)	112.8(85.6-133.8)	58.3(23.4-99.7)
Obstructive nephropathy	6	694.2(431.1-1336.7)	85.7(58.6-114.7)	88.5(51.0-122.1)
Reflux nephropathy	15	724.3(382.3-1075.7)	115.7(75.1-143.8)	69.9(51.3-91.4)
Multicystic dysplastic kidney disease	6	634.2(538.8-842.7)	133.1(104.4-154.0)	78.3(61.4-118.5)
Prune-belly syndrome	1	347.5	60.9	18.2
Glomerulonephritis	15	331.0(143.0-965.3)	111.3(81.3-137.0)	84.2(59.8-129.5)
Nephrotic syndrome	13	847.0(622.4-1190.8)	85.3(55.8-120.7)	110.0(40.4-167.2)
IgA nephropathy	7	1004.8(641.5-1339.8)	106.6(75.8-137.0)	71.6(42.3-84.3)
Lupus nephritis	4	567.7(443.7-745.7)	103.5(58.7-175.1)	31.0(14.6-56.1)
Purpura nephritis	2	235.9	122.9	107.6
ANCA vasculitis	1	193.0	106.9	128.8
PSGN	1	630.4	75.5	146.5
FSGS	1	747.8	133.1	44.6
Solidary kidney	3	1047.9	70.4	52.9

Data are medians (25th, 175th percentile); solidary kidney etiology: wilms' tumor post nephrectomy*1, congenital mesoblastic nephroma post nephrectomy*1, left duplex with obstructive nephropathy post nephrectomy*1

Table S2. Plasma complement factor H and related protein level in children with non-CAKUT group under different immunosuppressant therapy

	Prednisolone	Prednisolone+ cyclosporin	none
n	17	4	23
CFH	757.55(471.11-1011.96)	473.06(317.02-714.91)	630.35(221.26-1072.53)
CFHR2	93.25(69.61-121.46)	83.76(18.69-131.28)	111.27(75.84-137.05)
CFHR3	67.30(31.97-124.67)	86.35(34.17-228.04)	81.84(59.81-126.81)

Data are medians (25th, 75th percentile); No significant difference between three groups by Kruskal-Walis test

Table S3. Plasma level of CFH, CFHR2, CFHR3 vs. ABPM profile in the CAKUT group.

**ABPM**		**CAKUT (n=58)**			
		n	CFH	CFHR2	CFHR3
24 hr BP	Abnormal	9	697.4(392.8–1091.2)	115.7(109.6–173.5)	53.5(25.4–95.5)
	Normal	49	715.9(503.7–1030.0)	96.8(70.9–143.4)	62.1(41.3–93.3)
Daytime BP	Abnormal	5	744.5(301.1–1449.8)	116.4(93.0–180.0)	53.5(18.6–137.4)
	Normal	53	697.4(503.7–1009.2)	101.4(72.3–143.4)	60.5(39.1–90.8)
Nighttime BP	Abnormal	12	636.0(387.5–986.5)	125.5(83.0–173.7)	54.1(25.9–105.9)
	Normal	46	720.1(504.1–1048.2)	99.0(72.6–137.4)	67.2(41.0–92.4)
BP load	Abnormal	35	724.3(502.9–1047.9)	115.7(75.1–154.3)	60.5(37.8–91.4)
	Normal	23	618.5(364.6–1006.2)	96.8(70.1–133.6)	60.2(35.7–95.2)
Night dipping	Abnormal	30	710.8(472.8–1055.9)	114.3(70.3–152.9)	64.8(39.3–120.0)
	Normal	28	695.7(428.5–969.9)	102.5(75.4–134.1)	60.1(30.1–78.1)
ABPM profile	Abnormal	43	724.3(502.9–1047.9)	113.0(73.0–152.4)	60.1(37.8–95.2)
	Normal	15	618.5(347.5–1006.2)	96.8(71.5–133.6)	62.1(26.9–90.2)
Office BP	Abnormal	17	635.0(325.4–936.2)	106.4(77.9–151.9)	65.2(36.5–76.3)
	Normal	41	724.3(503.7–1062.5)	101.4(72.2–143.4)	60.1(36.8–96.0)

* $p < 0.05$ in abnormal and normal group by the Mann–Whitney U-test.

Table S4. Plasma level of CFH, CFHR2, CFHR3 vs. ABPM profile in the non-CAKUT group.

**ABPM**		**Non-CAKUT(n=44)**			
		n	CFH	CFHR2	CFHR3
24 hr BP	Abnormal	10	567.7(284.6–758.6)	126.2(87.3–138.9)	60.2(29.9–114.5)
	Normal	34	771.5(304.4–1171.3)	99.9(72.1–120.8)	84.2(59.0–129.0)
Daytime BP	Abnormal	6	567.7(400.3–668.1)	107.9(74.2–147.3)	54.0(23.2–75.8)*
	Normal	38	771.5(291.7–1097.0)	108.9(75.3–123.7)	84.2(56.8–129.0)*
Nighttime BP	Abnormal	12	567.7(228.2–721.2)	122.9(83.3–132.6)	60.2(30.6–104.3)
	Normal	32	819.5(330.0–1173.6)	99.9(75.1–121.5)	84.2(60.8–129.3)
BP load	Abnormal	23	609.4(312.8–853.7)	117.8(75.0–126.6)	63.4(31.4–110.0)*
	Normal	21	785.4(331.2–1193.1)	93.3(75.7–128.8)	107.1(69.5–150.6)*
Night dipping	Abnormal	28	612.9(244.1–881.1)	106.8(75.1–124.3)	77.5(57.7–121.9)
	Normal	16	881.2(351.4–1173.6)	117.8(77.3–134.4)	75.2(36.6–163.8)
ABPM profile	Abnormal	35	616.4(312.8–965.3)	106.9(75.5–123.0)	73.2(56.4–120.5)
	Normal	9	1016.9(428.4–1076.3)	120.5(72.4–146.1)	126.8(40.4–171.3)
Office BP	Abnormal	14	567.7(289.9–1055.3)	115.7(61.0–134.1)	65.3(44.1–138.3)
	Normal	30	771.5(305.3–1021.7)	99.9(75.7–123.1)	84.2(54.3–123.5)

Data are medians (25th, 75th percentile);* $p < 0.05$ in abnormal and normal group by the Mann–Whitney U-test.
